# The Efficacy of Treatment of Different Intervention Programs for Patellofemoral Pain Syndrome–A Single Blinded Randomized Clinical Trial. Pilot Study

**DOI:** 10.1100/tsw.2007.167

**Published:** 2007-08-24

**Authors:** Feazadeh Avraham, Saposhnik Aviv, Pnina Ya'akobi, Hava Faran, Zilla Fisher, Yael Goldman, Guy Neeman, Eli Carmeli

**Affiliations:** ^1^ “Raziel” Physical Therapy Department, Clalit Health Services, Netanya, Israel; ^2^ Physical Therapy Clinic, Stanley Sackler Faculty of Medicine, Tel Aviv University, Israel

**Keywords:** Patello-femoral pain syndrome, rehabilitation, knee, Israel

## Abstract

Patello-femoral pain syndrome (PFPS) is a common knee joint disability. The integration of hip soft tissue regimens are not always emphasized, although current literature implies that there is a significant relationship between the two and there is a lack of randomized clinical trials to substantiate this relationship in clinical practice. A randomized controlled assessor blinded trial was designed to explore different rehabilitation programs related to PFPS. The study was conducted at RAZIEL institute of physical therapy, Netania, Israel with a total of 30 consecutive patients (mean age 35y), diagnosed with PFPS. All patients were randomly allocated into 3 groups. Group I conventional knee rehabilitation program. Included quadriceps strengthening and Trans Electric Neuromuscular Stimulation (TENS). Group II hip oriented rehabilitation program. included stretching, Hip external rotators strengthening and TENS. Group III a combination of the two above programs. Pain and function were documented on initial of the program and again 3 weeks later, on the completion. Pain was assessed by a numeric visual analogue scale (VAS); function was assessed by Patello-femoral joint evaluation scale (PFJES) (0-100 points). At end of trial, all groups showed significant improvements in VAS and PFJES (p<0.0001); these improvements did not vary significantly between the 3 groups. The conclusions were that the explored different rehabilitation programs showed a similar beneficial effect.

## INTRODUCTION

Patello-femoral pain syndrome (PFPS) is a common knee joint disability, it accounts for 25% of all knee injuries treated in sports medicine clinics[1]. Symptoms usually seen are peripatellar and/or retropatellar pain, crepitus, giving way, stiffness and swelling[2]. Nonoperative treatment have successful results in resolving the syndrome ranging between 66% to 87%[3,4], although there is no agreement for a standard regimen. Conventional treatment usually includes quadriceps strengthening, patellar bracing and taping, soft tissue mobilization and stretching[5,6].

This treatment approach addressed the theory that patellar mal-tracking and/or mal-alignment is the key problem[7,8,9,]. Controversy exists regarding the relations between the knee alignment and the presence of symptoms[2,6]. Quadriceps strengthening and particularly the vastus medialis oblique is emphasized because it is believed that weakness may predispose for muscle imbalance and concomitant patellar stress. This assumption suffers from inconsistency in literature[10,11]. Recently, proximal stabilization of the lower limb has received more attention. Powers et al[12] suggested that the patellofmoral joint kinematics during weight bearing could be characterized as the femur rotating underneath the patella. The lack of control of femoral adduction and internal rotation caused by weak hip abductors and external rotators could be a primary cause for malalignment. These findings seem to be concurrent with a study by Ireland et al[13] which show 26% weaker hip abductors and 36% weaker hip external rotators in the comparison between symptomatic and nonsymptomatic females.

Muscle stretching as part of a regimen for PFPS is stressed out, especially the iliotibail band (ITB), due to its stressing effect on the lateral aspect of the patella and the lateral peripatellar tissues when over tight [5,7].

It is suggested that decreased hamstring flexibility correlates with PFPS[3,5,8,10]. The purpose of this study was to objectively evaluate 3 different PFPS rehabilitation programs. Two programs emphasizing an area for intervention and one program that combines the two, for the alleviation of PFPS.

## METHODS

*Participants*: Forty-two consecutive patients (mean age 35 years) were recruited to the study. All patients were diagnosed with PFPS by an orthopedic surgeon according to the following inclusion criteria's based on clinical findings[2]:

1. Positive sign (i.e., local pain) in patellofemoral gliding test.
2. Negetive McMurry test.
3. Full knee range of motion.
4. Anterior knee pain, related to prolonged sitting, climbing stairs, and descending stairs.
5. No relevant patellofemoral degenerative changes on imaging.
6. No history of knee trauma. All patients were randomly allocated into 3 groups (see Table 1).

*Outcome measures:* Pain and function were assessed by a blinded tester in the beginning of the program and 3 weeks at the end. Pain was assessed by a numerical visual analogue scale (VAS), 0 indicates no pain, and 10 indicates most severe pain. Everyday function was assessed by Patellofemoral evaluation scale[14] (0-100 points).

*Intervention:* Intervention program is showed in Table 1. The intervention program lasted 3 weeks, 2 treatments per week at the clinic, each treatment lasted for 30 minute. In addition, four home self-treatments were ordered to each patient during the remaining days of the week. Home self-treatment lasted 15 minutes and performed in parallel the clinic exercises. Each patient seemed ready to commence the program at home when he/she was able to correctly carry out the movement in the clinic with minimal verbal cues. As muscle strength and motor control improved, patients were instructed to increase the load time and repetitions.

*Quadriceps strengthening exercises:* 1. Straight leg raise (SLR). Patient lies on his back with shoes-off. The no symptomatic knee was flexed, foot placed on the bed. Symptomatic leg positioned in straight on the bed. During the exercise the hip was flexed into 30 degrees while keeping knee at full extension and ankle at full dorsi flexion. The limb was held for 10 seconds, lowered down to the bed for 10 seconds and repeated according to the exercise protocol. 2. Single leg squats- Patient stood on the symptomatic limb with shoes on. Contralateral limb was at 60 degrees in hip flexion and 90 degrees in knee flexion. Patient flexed the symptomatic knee into 30 degrees and flexed it back repeatedly. Patients were instructed to carryout the exercise according to their capability and rest at a ratio of 1:1.

*Hip External rotators strengthening:* The patient was lying on his/her symptomatic side. Non symptomatic limb remained straight. The symptomatic limb was flexed at the hip and knee into 90 degrees; in such manner the thigh is beyond the edge of the bed. In resting position the hip is placed in internal rotated with no resistance to gravity. At contraction, the patient externally rotated the hip into 30 degrees. The Physical therapist prevented any excess movements. As in previous exercises patients were instructed to carryout the exercise according to their capability and rest at ratio of 1:1.

*Hamstring stretching:* Patient was lying on bed, both limbs straight. The physical therapist flexes the hip of the symptomatic leg to 90 degrees. In this position the therapist extended the knee to the point when the patient said that he feels stretching sensation. The therapist held this position for 10 seconds and then relaxed the limb for 10 second, repeatedly.

*ITB stretching:* As recommended by Wang et al.,[15] Therapist stretched the limb up to the point when the patient said that he feels stretching sensation. The therapist held this position for 10 seconds and then relaxed the limb for 10 second, repeatedly.

*Tens:* After exercises, each patient received 15 minutes of sensory TENS for pain relieving. The instrument used was Compact TENS model BD03-0001. Four silicon electrodes 4*4 cm' were placed around the patella. Lectron 2 conductivity gel was used as a mediator. The instrument was set to 100 Hz and 150 ms.

## Statistics

Using Wilcoxon signed-ranks test, we compared pain before intervention to pain at completion of intervention and function before intervention to function at completion of intervention. Mann-Whitney test was used to test for difference between the outcomes of the groups. The level of significance was set at $P<0.05$.

**Table 1.**
**Group's intervention protocols**

| Group III | Group II | Group I |
|---|---|---|
| 3 minutes SLR | 3 minutes ITB stretching | 7.5 minutes SLR |
| 3 minutes Single leg squats. | 3 minutes Hamstring stretching | 7.5 minutes Single leg squats |
| 3 minutes ITB stretching | 9 minutes Hip external rotators strengthening | 15 minutes TENS |
| 3 minutes Hamstring stretching | 15 minutes TENS | |
| 3 minutes Hip external rotators strengthening | | |
| 15 minutes TENS | | |

## RESULTS

Out of 42 recruited patients 30 remained, 10 in each group. Twelve patients dropped out because of inconsistency in the program. All three groups showed significant improvement in both outcome measures (Figure 1, 2). The significant of group II and III showed more power than group I. No significant differences were found between groups in the outcome measures separately (Figure 3, 4).

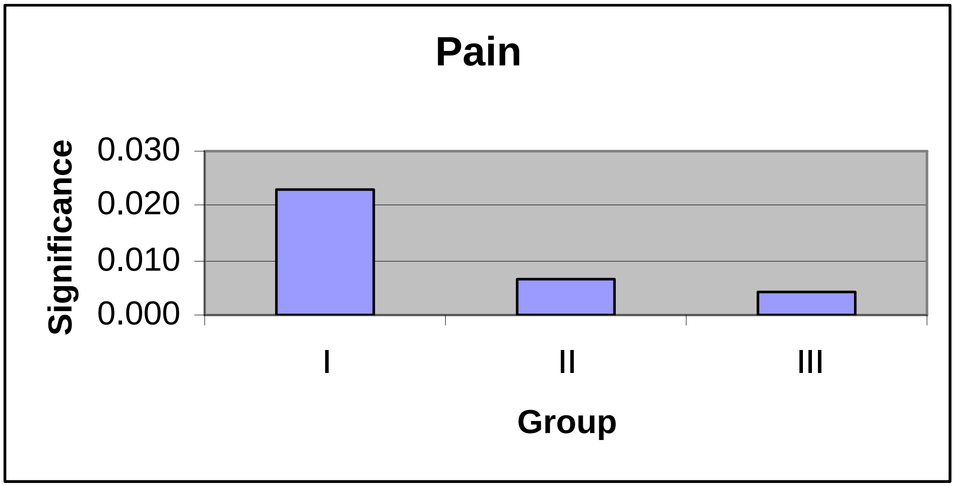

**FIGURE 1.** Pain changes.

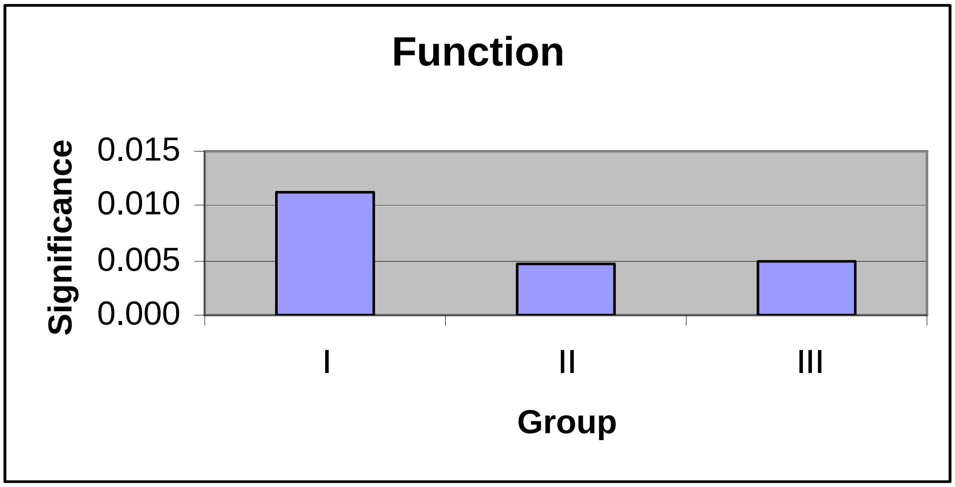

**FIGURE 2.** Function changes.

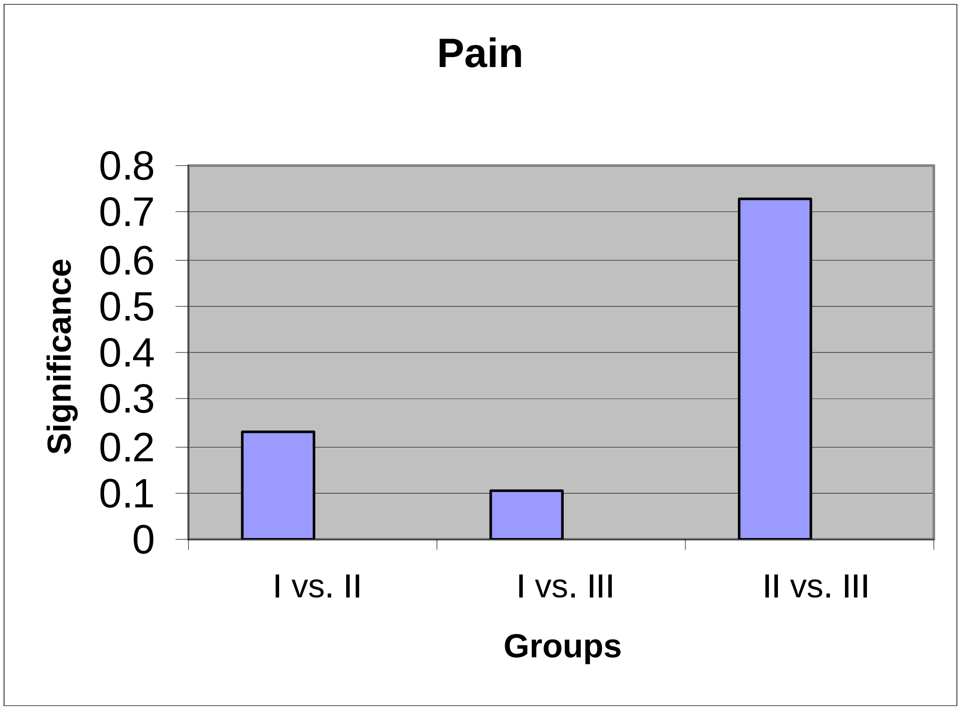

**FIGURE 3.** Pain variation between groups.

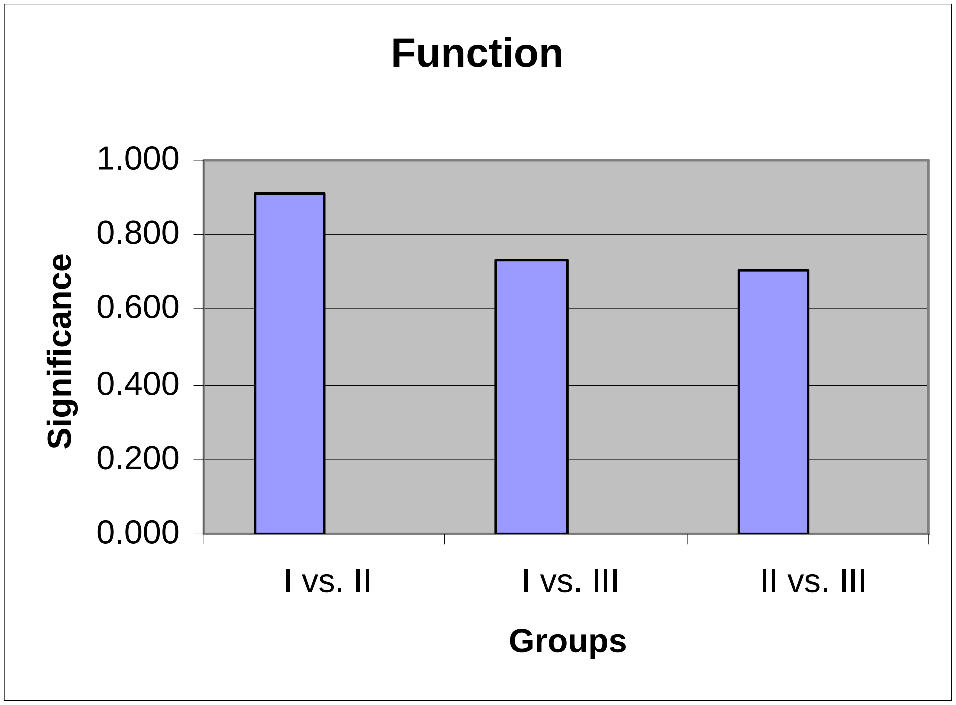

**FIGURE 4.** Function variation between groups.

Conventional PFPS intervention traditionally emphasizes quadriceps strengthening and/or quadriceps neuromuscular control training. The importance of hip strengthening and flexibility in the treatment of PFPS has received increased attention in the recent years. It is hypothesized that adequate pelvic-hip dynamic control, will help the patient to improve patellofemoral joint mechanics[5,13]. In the present study, all three groups showed similar beneficial effect related to the documented outcome measures.

Clark et al[3] conducted a randomized clinical trail to explore different physical therapy programs for anterior knee pain. Four groups where tested with 4 different intervention programs: (1) exercise, education, and taping; (2) taping and education; (3) exercise and education; (4) education alone. At three months VAS improved significantly in all patients, but these improvements did not vary significantly. At one year, patients who performed exercises had significantly lower pain scores than those who did not.

Tyler et al[5] showed, in a six weeks hip muscle oriented program, significant improvement in outcome measures of 66% of the participant. He attributed the improvement to iliopsoas strengthening and ITB flexibility. Since our study was conducted for a period of three weeks, it might be too early to search for significant differences. Nevertheless the different regimens are well known in clinical practice and a period of three weeks usually shows some improvements in pain and function.

Interestingly, the significance of the hip intervention group and combined interventions group were higher than the quadriceps intervention group, although found insignificant. This result may suggest that giving more emphasis to proximal stabilization in PFPS rehabilitation programs will help better alleviation of symptoms.

Several explanations may help to understand our results: 1) Time may be an important factor in alleviation of pain; since we did not have a placebo group it is not possible to reach any conclusions regarding the subject. 2) Formerly untrained patients may predispose to musculoskeletal pain. Exposing the patient to adequate exercising program and physical activity education may be an important factor in preventing musculoskeletal pain. 3) Relative rest may contribute to a self-healing process. In a review by Post [6] stated that it is important to control duration, intensity and frequency of activity, during PFPS rehabilitation program. It is more successful to keep the patients total activities below the level that triggers pain and tissue damage. 4) Sample size (n=30) might be too small to detect any significant differences between groups. 5) We lacked a control group of the home exercising program; and 6) inconsistency of a patient might influence the results.

Although the current study tried to explore different regimens, the hip program did not address all elements, such as hip abductors and hip flexors neuromuscular training; this area may be one of future research. Other factors that are important to consider is a larger sample size of participants, thus it will be possible to draw more substantial conclusions between groups, a placebo group is important to reach conclusion regarding the time component of rehabilitation. Adopting a tool to control the home exercising program will make the results more accurate.

## CONCLUSIONS

The current study explored different intervention protocols for the alleviation of PFPS. All 3 groups showed similar efficiency, with stronger significance to the groups who combined hip strengthening and stretching exercises. Future research may explore the integration of other hip muscles and soft tissues. Enlarging the sample size, creating tools for controlling the home exercise program and adding placebo group may attribute to reliability and validity of the research.
